# Comparison of long-term changes in peripapillary RNFL thickness between macula-on and macula-off rhegmatogenous retinal detachment

**DOI:** 10.1038/s41598-025-00799-5

**Published:** 2025-05-08

**Authors:** Jung-Tae Kim, Kee-Sup Park, Ka-Hyun Lee, Young-Hoon Lee, Min-Woo Lee

**Affiliations:** 1 Department of Ophthalmology, Konyang University College of Medicine, Daejeon, Republic of Korea; 2 Modoo’s Eye Clinic, Daejeon, Republic of Korea; 3 Department of Ophthalmology, Konyang University Hospital, #1643 Gwanjeo-dong, Seo-gu, Daejeon, Korea

**Keywords:** Rhegmatogenous retinal detachment, Retinal nerve fiber layer, Axial length, Diseases, Eye diseases, Retinal diseases

## Abstract

To compare postoperative changes in peripapillary retinal nerve fiber layer (pRNFL) thickness between macula-off and macula-on rhegmatogenous retinal detachment (RRD). Patients with RRD who had undergone a single, uncomplicated vitrectomy and been followed for ≥ 3 years postoperatively were included. Based on preoperative status, patients were categorized into a macula-on group (Group 1) and a macula-off group (Group 2). The baseline was established after complete gas dissipation from the vitreous cavity, followed by three additional examinations at 1-year intervals. In total, 62 eyes were analyzed: 30 in Group 1 and 32 in Group 2. Global pRNFL thicknesses in Group 1 were 100.0 ± 19.5, 99.4 ± 19.6, 98.4 ± 19.4, and 97.0 ± 20.3 μm at baseline, 1 year, 2 years, and 3 years, respectively (*P* = 0.001). In Group 2, the corresponding values were 99.6 ± 15.0, 96.2 ± 16.4, 95.4 ± 16.3, and 94.1 ± 17.6 μm (*P* < 0.001). Sectoral analysis showed statistically significant changes in the inferotemporal (*P* < 0.001) and inferonasal (*P* = 0.003) sectors in Group 2. The reduction rates of global pRNFL thickness were − 0.89 μm/y in Group 1 and − 1.81 μm/y in Group 2; these rates significantly differed between the groups (*P* = 0.026). Among RRD patients, pRNFL thickness gradually declined over time, with a more pronounced reduction in the macula-off group. A substantial decrease in inferior pRNFL thickness was observed in macula-off patients.

## Introduction

Rhegmatogenous retinal detachment (RRD) occurs when liquefied vitreous humor enters the subretinal space through retinal breaks, causing the separation of the retinal pigment epithelium layer from the sensory retinal layer. It is more prevalent in men than in women and is most commonly observed in individuals aged 20–60 years, although it can occur at any age^1,2^ Risk factors for RRD include myopia, lattice degeneration, inflammation, retinal injury, atopic dermatitis, and posterior vitreous detachment^3,4^ Surgical intervention is often required to treat RRD; it may involve procedures such as scleral buckling and vitrectomy, performed either alone or in combination depending on the specific case. These surgeries aim to achieve retinal reattachment by closing retinal breaks and relieving traction^5^.

Surgical treatment for RRD is typically highly successful in reattaching the retina^6^ However, visual prognosis after reattachment may be affected by several factors; one of the most critical is the macular detachment status prior to surgery^7^ Retinal reattachment can lead to microstructural changes in the retina, including alterations in the external limiting membrane, ellipsoid zone, and interdigitation zone at the macula, which may impact visual outcomes. These changes are often influenced by macular involvement during detachment^8,9^ Furthermore, RRD may induce changes in peripapillary retinal nerve fiber layer (pRNFL) thickness, even after successful reattachment^10^ It is hypothesized that more severe pRNFL damage progression occurs in macula-off RRD, where detachment ends to be more extensive in the posterior pole area. However, no studies have specifically evaluated this aspect.

The purpose of this study was to compare postoperative changes in pRNFL thickness between macula-off and macula-on RRD.

## Methods

### Patients

This retrospective, longitudinal, observational study adhered to the tenets of the Declaration of Helsinki, and the study protocol was approved by the Institutional Review Board/Ethics Committee of Konyang University Hospital, Daejeon, Republic of Korea (No. 2024-10-022). Patients who visited the retinal clinic between March 2015 and May 2024 were screened for inclusion. The Institutional Review Board/Ethics Committee of Konyang University Hospital waived the requirement for informed consent due to the retrospective study design. Patients with RRD who underwent a single, uncomplicated vitrectomy and had a minimum follow-up period of 3 years postoperatively were included. The surgeries were performed by two surgeons (Y.H.L. and M.W.L.). All patients underwent 25-gauge pars plana vitrectomy, and phacoemulsification with posterior chamber lens implantation was performed in patients with cataracts. The preexisting peripheral retinal breaks were used to drain the subretinal fluid after filling with perfluorocarbon liquid. Subsequently, fluid-air exchange was performed and endo photocoagulation was applied around the breaks in the peripheral retina. After PFCL removal, gas endo tamponade (C3F8 or SF6) was applied, and patients were advised to maintain a prone position for two weeks following the surgery. Patients who received silicone oil injections, underwent internal limiting membrane peeling or retinotomy during vitrectomy, which could affect pRNFL thickness measurements, or had concurrent epiretinal membrane or vascular diseases such as retinal vein occlusion were excluded. Based on preoperative status, patients were categorized into a macula-on group (Group 1) and a macula-off group (Group 2). The baseline was established after complete gas dissipation from the vitreous cavity, followed by three additional examinations at 1-year intervals. We excluded patients with a history of ocular diseases other than RRD, pathologic myopia with diffuse chorioretinal atrophy, and intraocular pressure > 21 mmHg after gas dissipation. During the follow-up period, we excluded not only patients with RRD-related complications such as epiretinal membrane but also those with retinal abnormalities that were less directly related to RRD, including diabetic retinopathy, retinal vein occlusion, or age-related macular degeneration.

### pRNFL thickness measurements

A skilled examiner measured pRNFL thickness using spectral-domain optical coherence tomography (Spectralis; Heidelberg Engineering, Heidelberg, Germany). Scans were obtained in a circular scanning pattern with a 3.5 mm diameter around the optic nerve head, capturing pRNFL thickness in both global and sectoral areas, including the temporal, superotemporal, superonasal, nasal, inferonasal, and inferotemporal sectors. Scans with a quality score below 15, as well as images showing decentration, misalignment, or severe segmentation errors, were excluded from the analysis.

### Statistical analysis

Baseline demographic characteristics and ocular parameters were compared between groups using independent t-tests, while categorical variables were analyzed with Chi-squared tests. Linear mixed models were utilized to assess changes in pRNFL thickness over time within each group, including the rate of thickness reduction, with a random intercept at the eye level. Generalized linear mixed models were used to identify factors associated with changes in pRNFL thickness over time. Statistical analyses were performed with SPSS software (version 18.0; IBM Corp., Armonk, NY, USA).

## Results

### Demographic characteristics

**Fig. 1:**
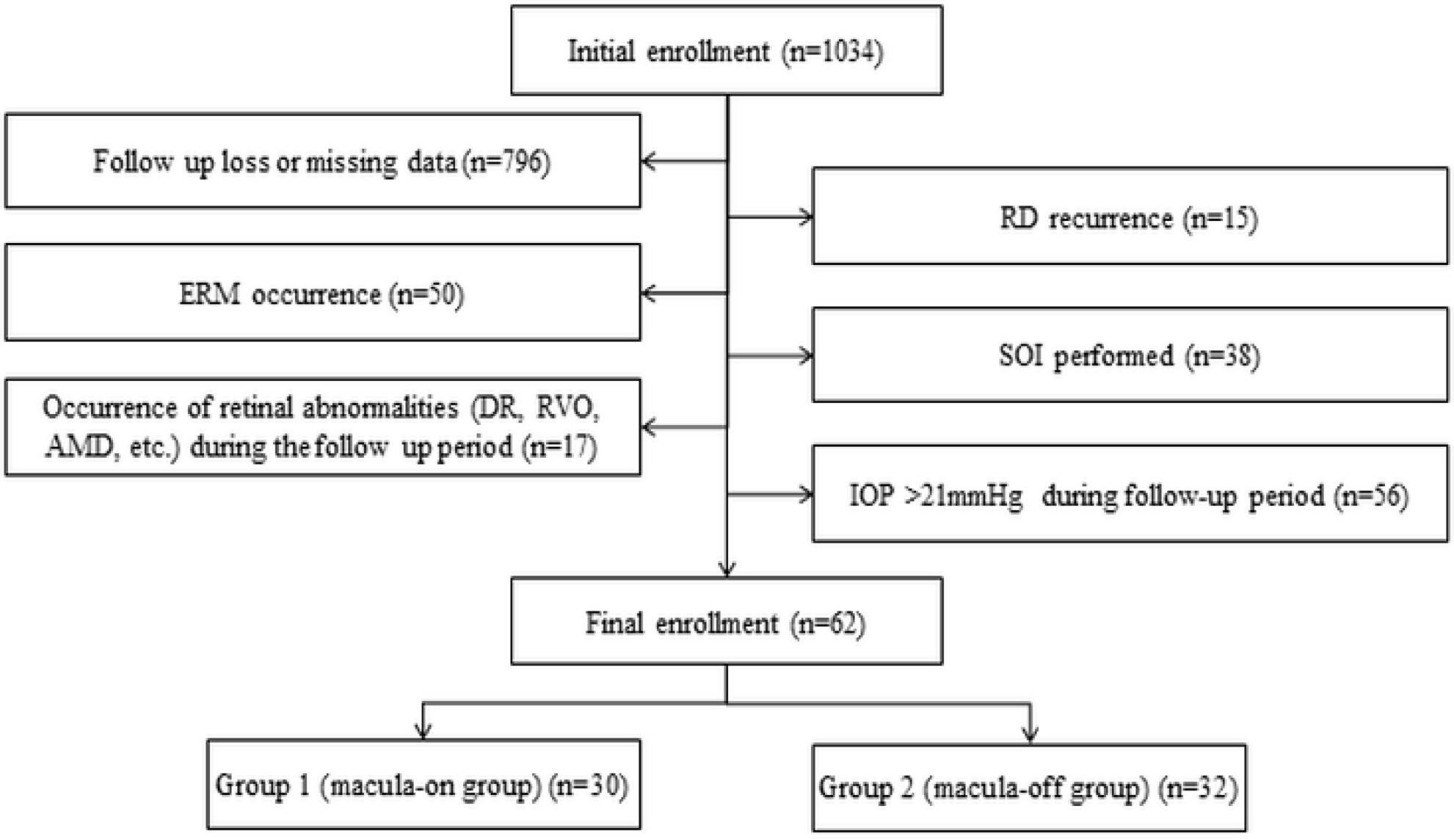
Flow diagram showing number of subjects included in the study population. *RD* retinal detachment, *ERM* epiretinal membrane, *SOI* silicone oil injection, *DR* diabetic retinopathy, *RVO* retinal vein occlusion, *AMD* age-related macular degeneration, *IOP* intraocular pressure.

In total, 62 eyes were included in the study; 30 in Group 1 and 32 in Group 2 (Fig. 1). The mean ages were 48.6 ± 14.8 years in Group 1 and 48.6 ± 13.9 years in Group 2 (*P* = 0.999) (Table 1). The mean duration from the diagnosis of RRD to surgery was 3.3 ± 1.5 days in Group 1 and 3.1 ± 1.4 days in Group 2 (*P* = 0.821). The mean times from surgery to baseline were 9.7 ± 11.7 months in Group 1 and 7.5 ± 5.4 months in Group 2 (*P* = 0.344). The mean best-corrected visual acuity significantly differed between the groups: 0.11 ± 0.22 in Group 1 and 0.38 ± 0.35 in Group 2 (*P* = 0.001). The mean axial lengths were 25.8 ± 2.1 mm in Group 1 and 25.7 ± 1.8 mm in Group 2 (*P* = 0.298). Other factors, including sex, spherical equivalent, intraocular pressure, symptom duration, and location of retinal elevation, Showed no significant differences between the groups.

**Table 1:** Baseline demographic characteristics.

	Group 1 (*n* = 30)	Group 2 (*n* = 32)	*P*-value
Age (years, mean ± SD)	48.6 ± 14.8	48.6 ± 13.9	0.999
Sex (male, %)	11 (36.7)	16 (50.0)	0.343
Laterality (right, %)	15 (50.0)	12 (37.5)	0.264
Diabetes (n, %)	0 (0.0)	3 (9.4)	0.096
Hypertension (n, %)	3 (10.0)	7 (21.9)	0.247
Symptom duration (days, mean ± SD)	8.6 ± 9.8	10.2 ± 11.5	0.571
Location of retinal elevation (n, %)			0.351
Superior	16 (53.3)	12 (37.5)	
Inferior	4 (13.3)	6 (18.8)	
Both	10 (33.3)	14 (43.8)	
Lens status (pseudophakic, %)	16 (53.3)	18 (56.3)	0.933
SE (diopters, mean ± SD)	-3.28 ± 3.74	-2.99 ± 2.58	0.244
IOP (mmHg, mean ± SD)	14.2 ± 3.6	13.8 ± 3.1	0.407
Axial length (mm, mean ± SD)	25.8 ± 2.1	25.7 ± 1.8	0.298
BCVA (logMAR, mean ± SD)	0.11 ± 0.22	0.38 ± 0.35	**0.003**

*SE* spherical equivalent, *IOP* intraocular pressure, *BCVA* best-corrected visual acuity. Values in boldface (*P* < 0.050) are statistically significant.

### pRNFL thickness at each visit

**Fig. 2:**
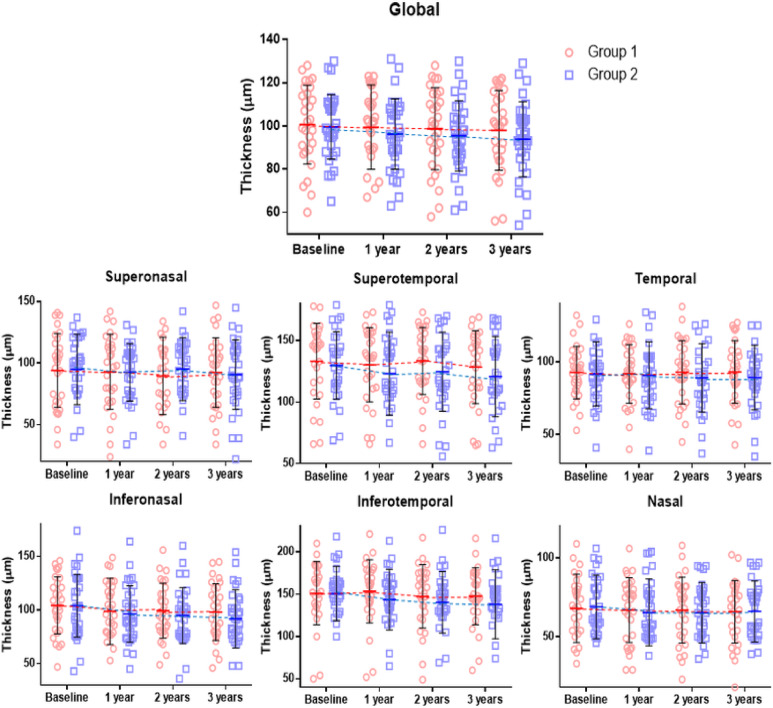
Scatter plots and line graphs showing means and standard deviations of peripapillary retinal nerve fiber layer thickness at each visit.

In Group 1, global pRNFL thicknesses were 100.0 ± 19.5, 99.4 ± 19.6, 98.4 ± 19.4, and 97.0 ± 20.3 μm at baseline, 1 year, 2 years, and 3 years, respectively (*P* = 0.001), which showed a significant reduction over time (Table 2). In Group 2, global pRNFL thicknesses were 99.6 ± 15.0, 96.2 ± 16.4, 95.4 ± 16.3, and 94.1 ± 17.6 μm at baseline, 1 year, 2 years, and 3 years, respectively (*P* < 0.001), which also showed a significant reduction over time. Although most sectors in both groups showed a trend of decreasing thickness, statistically significant reductions were observed only in the inferotemporal (*P* < 0.001) and inferonasal (*P* = 0.003) sectors in Group 2 (Fig. 2).

**Table 2:** Peripapillary retinal nerve fiber layer thickness in each group at each visit.

	Group 1	Group 2	*P*-value*
Global			
Baseline	100.0 ± 19.5	99.6 ± 15.0	0.137
First year	99.4 ± 19.6	96.2 ± 16.4	
Second year	98.4 ± 19.4	95.4 ± 16.3	
Third year	97.0 ± 20.3	94.1 ± 17.6	
P-value^†^	**0.001**	**< 0.001**	
Sector			
Temporal			
Baseline	92.5 ± 17.9	91.8 ± 22.0	0.595
First year	91.7 ± 20.2	90.8 ± 23.1	
Second year	92.4 ± 21.8	89.1 ± 23.5	
Third year	92.7 ± 21.6	89.2 ± 22.1	
P-value^†^	0.356	0.300	
Superotemporal			
Baseline	133.4 ± 30.4	125.8 ± 35.5	0.854
First year	130.3 ± 30.2	123.2 ± 33.7	
Second year	129.9 ± 26.7	121.7 ± 32.1	
Third year	127.9 ± 30.9	120.5 ± 32.8	
P-value^†^	0.109	0.082	
Superonasal			
Baseline	93.4 ± 31.8	94.9 ± 28.6	0.261
First year	93.0 ± 30.6	92.6 ± 23.3	
Second year	92.7 ± 31.3	92.2 ± 25.6	
Third year	91.6 ± 30.4	91.4 ± 28.3	
P-value^†^	0.276	0.174	
Nasal			
Baseline	67.1 ± 23.1	68.9 ± 20.1	0.470
First year	66.9 ± 20.5	65.4 ± 21.2	
Second year	66.7 ± 21.1	65.2 ± 19.2	
Third year	64.8 ± 21.8	64.9 ± 19.2	
P-value^†^	0.172	0.077	
Inferonasal			
Baseline	103.3 ± 28.4	104.0 ± 29.3	0.933
First year	98.8 ± 31.0	96.4 ± 26.4	
Second year	99.0 ± 26.1	94.9 ± 26.1	
Third year	97.1 ± 27.6	91.1 ± 28.1	
P-value^†^	0.215	**0.003**	
Inferotemporal			
Baseline	149.7 ± 38.8	150.8 ± 32.1	0.254
First year	148.2 ± 37.3	143.6 ± 35.8	
Second year	146.7 ± 38.1	140.3 ± 36.4	
Third year	145.6 ± 36.5	135.3 ± 42.3	
P-value^†^	0.059	**< 0.001**	

Values in boldface (*P* < 0.050) are statistically significant. *Independent t-test for baseline values. ^†^Linear mixed model.

Additionally, we performed the analysis after excluding patients with an axial length greater than 26.5 mm in each group, resulting in 22 in Group 1 and 25 in Group 2. The global pRNFL thicknesses were 102.4 ± 18.3, 101.7 ± 20.0, 101.3 ± 19.2, and 100.0 ± 18.9 μm in Group 1, and 101.7 ± 15.8, 99.6 ± 16.1, 98.2 ± 16.5, and 97.5 ± 15.7 μm in Group 2, respectively. Both groups showed a significant decrease in RNFL thickness over time (Group 1, *P* = 0.049; Group 2, *P* < 0.001), but the rate of decrease in Group 2 was significantly greater (*P* = 0.041).

### Reduction rate and factors associated with changes in pRNFL thickness

The rates of reduction in global pRNFL thickness were − 0.89 μm/y in Group 1 and − 1.81 μm/y in Group 2, indicating a significant difference between the groups (*P* = 0.026) (Table 3). In terms of sectoral thickness, Group 2 exhibited a greater reduction tendency compared with Group 1, although a statistically significant difference between the groups was identified only in the inferotemporal sector (*P* < 0.001).

**Table 3:** Rates of change in peripapillary retinal nerve fiber layer thickness, calculated using linear mixed models.

	Group 1	Group 2	^*^P-value
Global	-0.89 (-1.43 to -0.35)	-1.81 (-2.40 to -1.21)	**0.026**
Sector			
Temporal	0.55 (-0.65 to 1.75)	-0.76 (-2.23 to 0.71)	0.169
Superotemporal	-1.12 (-2.49 to 0.25)	-1.33 (-2.83 to 0.17)	0.836
Superonasal	-0.85 (-2.39 to 0.69)	-1.20 (-2.96 to 0.56)	0.569
Nasal	-0.62 (-1.53 to 0.29)	-0.91 (-1.92 to 0.10)	0.714
Inferonasal	-1.36 (-3.53 to 0.82)	-3.20 (-5.23 to -1.17)	0.108
Inferotemporal	-1.63 (-3.32 to 0.06)	-3.64 (-5.58 to -1.72)	**< 0.001**

Values in boldface (*P* < 0.050) are statistically significant. ^*^Interaction between group and duration in linear mixed models.

In univariate analysis, significant factors associated with changes in pRNFL thickness included axial length (estimate = -3.45, *P* = 0.003), final best-corrected visual acuity (estimate = -20.72, *P* = 0.030), and time (estimate = -1.37, *P* < 0.001), with time indicating whether the change in pRNFL thickness is significant with each passing year (Table 4). Multivariate analysis identified axial length (estimate = -3.38, *P* = 0.003) and time (estimate = -1.18, *P* < 0.001) as significant factors. In the group-based analysis, axial length was not significantly associated with changes in pRNFL thickness in Group 1 (estimate = -2.56, *P* = 0.201), whereas a significant association was found in Group 2 (estimate = -5.24, *P* < 0.001).

**Table 4:** Linear mixed-effect model determination of factors associated with changes in peripapillary retinal nerve fiber layer thickness.

	Univariate		Multivariate	
	Estimate (95% CI)	*P* value	Estimate (95% CI)	*P* value
Age	0.17 (-0.14 to 0.48)	0.281		
Sex	-4.42 (-13.28 to 4.44)	0.323		
Symptom duration	0.31 (-0.11 to 0.72)	0.143		
Baseline BCVA	-4.55 (-18.34 to 9.25)	0.512		
IOP	0.20 (-1.13 to 1.53)	0.760		
AXL	-3.45 (-5.65 to -1.26)	**0.003**	-3.38 (-5.57 to -1.19)	**0.003**
Final BCVA	-20.72 (-39.36 to -2.07)	**0.030**	-11.88 (-29.25 to 5.49)	0.175
Time	-1.37 (-1.78 to -0.96)	**< 0.001**	-1.18 (-1.72 to -0.65)	**< 0.001**

*BCVA* best-corrected visual acuity, *IOP* intraocular pressure, *AXL* axial length. Values in boldface (*P* < 0.050) are statistically significant.

## Discussion

In vitrectomized eyes, changes in oxygen distribution within the vitreous cavity can impact the extracellular matrix of the trabecular meshwork, potentially reducing aqueous outflow and increasing the risk of glaucoma^11,12^ Additionally, RRD may contribute to inner retinal damage through anterograde degeneration^13^ High myopia, a common and significant risk factor for RRD, can also influence changes in pRNFL thickness^14^ Thus, it is important to carefully analyze pRNFL thickness in patients who have undergone vitrectomy for RRD. Although the extent of retinal detachment may lead to varying degrees of pRNFL damage, no previous studies have provided a longitudinal analysis of this phenomenon. In this study, we performed a longitudinal and comparative analysis of changes in pRNFL thickness over time between macula-on and macula-off RRD cases. Our findings demonstrated a significant decrease in pRNFL thickness over time in both groups; a more rapid reduction was evident in patients with macula-off RRD patients. Notably, the inferior sectors exhibited the greatest thickness reduction in patients with macula-off RRD. Such reduction in pRNFL thickness among patients with RRD showed a significant association with axial length.

Both groups in this study showed a significant reduction in pRNFL thickness over time. This reduction may be associated with long axial lengths in both cohorts. Lee et al.^14^ reported that highly myopic eyes experienced a significantly greater decrease in pRNFL thickness over 2 years relative to normal eyes, with reduction rates of -0.95 μm /y in individuals aged 30 to 39 years, -1.70 μm/y in those aged 40 to 49 years, and − 1.69 μm/y in those aged 50 to 59 years. They explained this reduction to globe elongation, which leads to mechanical stretching and thinning of the retina. The patients in our study had long axial lengths approaching high myopia, which may have contributed to the observed reduction in pRNFL thickness. Although direct comparison is challenging due to the use of different optical coherence tomography devices and the shorter axial lengths in our study, the reduction rate in Group 1, at -0.89 μm/y, appears to be slightly lower than that reported in the previous study on highly myopic patients. This finding suggests that the impact of RRD on changes in pRNFL thickness is smaller in Group 1.

The rate of pRNFL thickness reduction was significantly greater in Group 2 than in Group 1. This finding suggests that, because other factors (e.g., axial length) did not differ between the groups, macula-off RRD contributes to a more pronounced reduction in pRNFL thickness. Faude et al.^13^ reported that photoreceptor degeneration is accompanied by a substantial loss of ganglion cell axons and the degeneration of numerous ganglion cells in the inner layers of the detached retina. Consequently, pRNFL thickness may be influenced by anterograde degeneration of the second and third neurons in the area of the detached retina^10^ The faster rate of pRNFL reduction observed in Group 2, which has a relatively larger area of retinal detachment around the posterior pole compared with Group 1, is therefore expected. Additionally, the significant thickness reduction observed in Group 2 during the first year may be related to the resolution of postoperative edema and the decrease in Muller cell proliferation, which typically increases immediately after surgery. Further studies are needed to confirm this hypothesis.

Although most sectoral thicknesses tended to decrease over time, these changes were not statistically significant in either group, possibly due to the small sample size. However, the inferior sectors in Group 2 demonstrated a statistically significant and pronounced decrease over time. This observation may be related to the anatomical structure of the lamina cribrosa, through which the nerve fibers pass. Compared with other quadrants, the inferior portions of the lamina cribrosa contain larger pores and provide less connective tissue support for ganglion cell axons^15^ Larger laminar pores accommodate more nerve fiber bundles and require greater metabolic resources^16^ Consequently, this increased metabolic demand renders the inferior sector more susceptible to various types of damage^17^ Although fewer patients in this study had RRD in the inferior region compared with the superior region, the inferior sector exhibited a more pronounced reduction in thickness. This finding suggests that the anatomical structure of the lamina cribrosa plays a more central role in sectoral pRNFL thickness reduction than the location of the retinal detachment.

In patients with RRD, axial length was significantly associated with changes in pRNFL thickness, consistent with the previous study reporting thinner pRNFL in cases of high myopia^14^ As axial length increases, there is a tendency for continued elongation over time^18^ This stretching effect may lead to further thinning of the pRNFL. Notably, in the subgroup analysis, axial length was not significantly associated with changes in pRNFL thickness in Group 1, whereas a significant association was observed in Group 2. Changes in pRNFL thickness in macula-off RRD patients appear to be more sensitive to axial length compared to such changes in macula-on RRD patients. This sensitivity may be attributed to the larger area of retinal detachment at the posterior pole in macula-off patients, making the pRNFL more vulnerable to damage from the stretching effect. Further research is needed to elucidate the precise mechanisms involved.

Several studies have reported results different from ours^19–21^ Hwang et al.^20^ reported that no pRNFL thinning or thickening was observed at 3 years following surgery. This difference from our study can be due to the following reasons. First, the axial length of patients included in this study is longer compared to the previous study, which may have contributed to the more pronounced pRNFL thinning observed. Second, while the previous study reported no significant difference in pRNFL changes between the macula-off and macula-on groups, the sample size in that study was relatively small, with only 8 patients included in the macula-off group. Most importantly, the baseline measurement in the previous study was taken before surgery, whereas in this study, the baseline was defined as the point after the complete disappearance of intraocular gas. When examining only the postoperative period in the previous study, the trend of pRNFL thinning appears similar to that observed in our study. Thus, the results of the two studies are not contradictory. It is believed that the differences in the results of other studies, compared to ours, were also due to variations in inclusion criteria, such as differences in baseline time points or the inclusion of patients who received silicone oil injections^19,21^.

Our study had several limitations. First, its retrospective design inevitably introduced selection bias. Second, the number of patients was limited due to strict inclusion and exclusion criteria. Due to the limited number of cases, a small number of diabetic patients were included in Group 2. Although they did not progress to diabetic retinopathy during the follow-up period, their inclusion may have introduced some degree of bias. Third, although we enrolled patients without pRNFL defects or a history of intraocular pressure exceeding 21 mmHg, we could not completely rule out the possibility of including patients with preperimetric glaucoma at baseline. Fourth, while the axial lengths of the included patients were close to high myopia, reflecting actual clinical situations, this presents challenges in isolating the effects of RRD alone on changes in pRNFL thickness. Although similar results were observed in the analysis excluding high myopia patients with an axial length greater than 26.5 mm in this study, further research with a larger sample size is needed. Although the surgical procedures and methods were not different between the two surgeons, the inability to exclude bias due to inter-surgeon variability is another limitation of this study. The strength of our study lies in its longitudinal comparative analysis of changes in pRNFL thickness between macula-on and macula-off cases, a topic that has not been previously reported.

In conclusion, patients with RRD who underwent vitrectomy and experienced no additional complications showed a gradual decrease in pRNFL thickness over time; a more pronounced reduction was evident in those with macula-off RRD. Although more patients had superior area detachments relative to inferior detachments, the greatest reduction in inferior RNFL thickness was observed in macula-off patients. This finding is believed to result from the anatomical structure of the lamina cribrosa. Furthermore, changes in pRNFL thickness demonstrated a significant association with axial length; this association was stronger in macula-off patients. Physicians should consider these changes in pRNFL thickness when evaluating ophthalmic diseases where pRNFL measurements are important in patients who have undergone RRD surgery.

## Data Availability

The datasets used and/or analyzed during the current study available from the corresponding author on reasonable request.
